# Inactivation of necroptosis-promoting protein MLKL creates a therapeutic vulnerability in colorectal cancer cells

**DOI:** 10.1038/s41419-025-07436-z

**Published:** 2025-02-20

**Authors:** Peijia Jiang, Sandhya Chipurupalli, Byong Hoon Yoo, Xiaoyang Liu, Kirill V. Rosen

**Affiliations:** Departments of Pediatrics & Biochemistry and Molecular Biology, Dalhousie University, Halifax, NS Canada

**Keywords:** Colon cancer, Necroptosis

## Abstract

Mortality from colorectal cancer (CRC) is significant, and novel CRC therapies are needed. A pseudokinase MLKL typically executes necroptotic cell death, and MLKL inactivation protects cells from such death. However, we found unexpectedly that MLKL gene knockout enhanced CRC cell death caused by a protein synthesis inhibitor homoharringtonine used for chronic myeloid leukemia treatment. In an effort to explain this finding, we observed that MLKL gene knockout reduces the basal CRC cell autophagy and renders such autophagy critically dependent on the presence of VPS37A, a component of the ESCRT-I complex. We further found that the reason why homoharringtonine enhances CRC cell death caused by MLKL gene knockout is that homoharringtonine activates p38 MAP kinase and thereby prevents VPS37A from supporting autophagy in MLKL-deficient cells. We observed that the resulting inhibition of the basal autophagy in CRC cells triggers their parthanatos, a cell death type driven by poly(ADP-ribose) polymerase hyperactivation. Finally, we discovered that a pharmacological MLKL inhibitor necrosulfonamide strongly cooperates with homoharringtonine in suppressing CRC cell tumorigenicity in mice. Thus, while MLKL promotes cell death during necroptosis, MLKL supports the basal autophagy in CRC cells and thereby protects them from death. MLKL inactivation reduces such autophagy and renders the cells sensitive to autophagy inhibitors, such as homoharringtonine. Hence, MLKL inhibition creates a therapeutic vulnerability that could be utilized for CRC treatment.

## Introduction

20% of patients with colorectal cancer (CRC) have metastatic disease at the time of diagnosis, and 40% develop metastases after localized disease treatment [1]. Five-year survival of patients with metastatic CRC is below 20% [1]. Hence, novel CRC therapies are needed.

Necroptosis is programmed necrosis driven by a protein kinase MLKL [2]. When activated, MLKL polymerizes, forms pores in the plasma membrane and thus kills the cell [2]. We noticed unexpectedly that a small molecule MLKL inhibitor necrosulfonamide [3] or MLKL gene knockout (KO) reduce, rather than promote, CRC cell survival. Hence, we tested whether MLKL inactivation might represent a novel approach for CRC treatment.

We found that CRC cell death caused by MLKL inactivation is enhanced by homoharringtonine (HHT), a drug used to treat chronic myelogenous leukemia [4]. We found that MLKL inactivation and HHT cooperate in blocking the ESCRT machinery-dependent basal autophagy of CRC cells and kill them by a form of cell death termed parthanatos. HHT is a ribosome inhibitor [5], and such inhibitors trigger ribotoxic stress response, a chain of events that activate protein kinase p38 MAP kinase (p38MAPK) which often triggers cell death-promoting signals [6].

Autophagy is a process of degradation of the cellular content mediated by the double-membrane vesicles termed autophagosomes [2]. One of the functions of autophagy is to promote cell survival by eliminating the toxic cellular content, including damaged proteins and organelles [7]. Endosomal sorting complexes required for transport (ESCRTs) mediate the formation of numerous types of vesicles, including autophagosomes [8] and promote autophagy via several mechanisms [9, 10].

Parthanatos is a form of programmed necrosis triggered by hyperactivation of poly(ADP-ribose) polymerase 1, an enzyme that promotes the formation of poly(ADP-ribose) [11]. Excessive poly(ADP-ribose) levels cause chromosomal DNA degradation and cell death [11].

We found that MLKL inactivation renders CRC cell autophagy strongly dependent on the presence of the ESCRT-I complex component VPS37A [8] and that in the absence of MLKL, HHT activates p38MAPK and thus prevents VPS37A from supporting autophagy. As a result of autophagy inhibition caused by the combined effect of MLKL inactivation and HHT, CRC cells die by parthanatos. Moreover, we found that a small molecule MLKL inhibitor necrosulfonamide and HHT strongly cooperate in reducing the ability of CRC cells to form tumors in mice. Hence, pharmacological MLKL inhibition combined with HHT treatment is a potential novel approach for CRC therapy.

## Materials and methods

### Cell culture

Cell lines DLD1, HCT116, LS180, SW480 and 293 T were cultured in DMEM (GIBCO) and 10% Fetal Bovine Serum (FBS) (Sigma), 100 U/ml penicillin (GIBCO), 100 μg/ml streptomycin (GIBCO), 0.29 mg/ml L-glutamine (GIBCO). Lack of mycoplasma contamination in all cells was established as published [12].

### Reagents

The following reagents were from Cell Signaling Technology, Danvers, MA. Bafilomycin A1 (catalogue# 54645S), Z-VAD(OMe)-FMK (catalogue# 60332S), Olaparib (catalogue# 93852S), SB203580 (catalogue# 5633S). The following other reagents were used. Vemurafenib, Santa Cruz Biotechnology, Dallas TX, USA (catalogue# CAS 918504-65-1), selumetinib, Cedarlane Labs, Burlington ON, Canada (catalogue# ENZ-CHM184-0050), pictilisib, Cedarlane Labs (catalogue# HY-50094-10MG), lapatinib Selleckchem, Houston, TX, USA (catalogue# S1028), Lipofectamine™ 3000 Transfection Reagent, Thermo Fisher Scientific Waltham, MA, USA (catalogue# L3000008), Tat-BECN1 peptide, Selleckchem Houston, TX, USA (catalogue# S8595), scrambled peptide, MedChemExpress, Monmouth Junction, NJ, USA (catalogue# HY-P4106).

### Antibodies

**Fig. 1:**
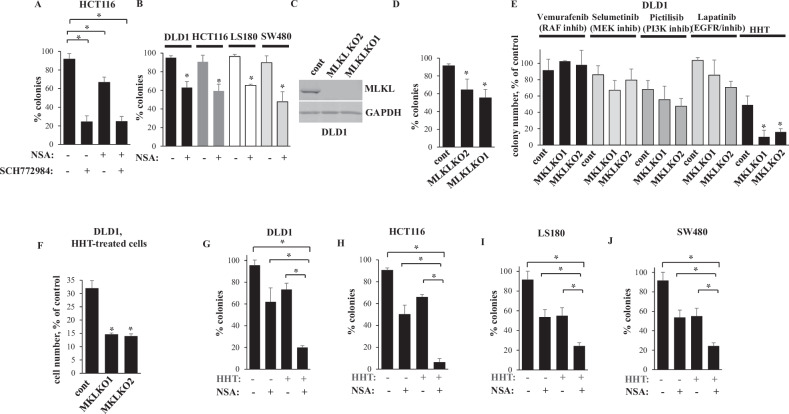
MLKL inactivation cooperates with HHT in reducing CRC cell clonogenic survival. **A** HCT116 cells were treated with DMSO (−) or 1 μM SCH 772984 (SCH) and/or 3 μM NSA (+), and colonies formed by the cells were counted. **B** Indicated cell lines were treated with DMSO (−) or 1 μM NSA (+), and colonies formed by the cells were counted. **C** Control cells (cont) or MLKL-deficient cells MLKL KO1 and MLKL KO2 were tested for MLKL levels by western blotting. GAPDH served as a loading control. **D** Cells generated in **C** were assayed as in **A**. **E** Indicated cell lines were treated or not with 5 μM vemurafrnib, 1 μM selumetinib, 1 μM pictilisib, 1 μM lapatinib or 10 ng/ml HHT and colonies formed by the cells were counted. **F** Indicated cell lines were treated or not with 10 ng/ml HHT for 96 h and counted. **G**–**J** Indicated cell lines were treated (+) or not (−) with 1 μM NSA and 10 ng/ml HHT, and colonies formed by the cells were counted. The data represent % of colony number formed by the untreated cells (**A**, **B**, **D**, **E**, **G**–**J**) or % of untreatded cells (**F**). The data in **A**, **G**–**J** are the average of the triplicates plus SD, and each experiment was repeated twice with similar results. The data in **E** are the average of two independent experiments plus SD. The data in **B** and **D** and **F** are the average of three independent experiments plus SD. *p value < 0.05.

The following antibodies used for western blotting were from Cell Signaling Technology, Danvers, MA, USA. Anti- LC3B (D11) (catalogue# 3868S), anti-DR4 (D9S1R) (catalogue# 42533S) anti-DR5 (D4E9) (catalogue# 8074S), anti-MLKL (D2I6N) (catalogue# 14993S), anti-alpha-tubulin (DM1A) (catalogue# 3873S), anti-poly/Mono-ADP Ribose (D9P7Z) (catalogue# 89190S), anti-p38 (catalogue# 9212), phospho-p38 (catalogue# 4511). Anti-VPS37A was from Proteintech, Rosemont, IL, USA (catalogue# 11870-1-AP). Western blotting experiments shown in Figs. 1C, 3C, 5A, B, 6A, C and in Supplementary Fig. 2A, B were performed twice with similar results. Western blotting experiments shown in Figs. 3A, 4A, B, D, 3B, 5C–F, 6B and 8F were performed three times with similar results.

### MLKL gene knockout by CRISPR

sgRNA-encoding cDNAs targeting exon 2 (TTGAAGCATATTATCACCCT) or exon 9 (TTAGCTTTGGAATCGTCCTC) of the MLKL gene were generated by use of CHOPCHOP web tool version 3 [13] and cloned into pX459 expression vector. DLD-1 cells were transfected with 2ug of pX459 encoding each sgRNA cloned pX459 for 1 day as described [14], the cells were treated with 3 μg/ml of puromycin for 2 days, and clonal cell lines were isolated by limiting dilution and analyzed for MLKL expression by western blotting. A clone that still expressed MLKL was chosen as a control clone, and clones that did not show MLKL expression in the case of each sgRNA were selected as MLKL-deficient clones.

### RNA interference

Small interfering (si)RNAs were used as described [15]. In the case of VPS37A knockdown experiments, the control and VPS37A siRNAs were from Thermo Fisher Scientific (Waltham, MA, USA). Silencer™ Select Negative Control No. 1 siRNA (cat # 4390843) served as a control RNA. The sequences of VPS37A siRNA1 and 2, respectively, were CGACAAAGAUGACUUAGUATT (cat #s44037) and CGACAUCACUUAAUGGAUATT (cat# s 44038). In the case of ATG12 knockdown experiments, the control and ATG12 siRNAs were from Horizon Discovery (Cambridge, UK). The sequence of the control ON-TARGETplus non-targeting RNA (cat # D-001810-01) was UGGUUUACAUGUCGACUAA. The sequences of ATG12 siRNA1 and 2, respectively were GGGAAGGACUUACGGAUGU (cat # J-010212-08) and GCAGUAGAGC GAACACGAA (cat # 010212-07).

### Drug treatment

Fo the clonogenic survival assay, in the case of MLKL KO clones, cells were plated on day 1, treated with a drug on day 2, and the medium was changed to drug-free medium on day 5. In the case of treatment of cells with NSA, HHT or other drugs described in the study, cells were plated on day 1, treated with NSA on day 2 to ensure that MLKL is inhibited before the cells are treated further, the second drug was added on day 3, and the medium was changed to drug-free medium on day 6 in the case of SCH 772984 and on day 7 in the case of other drugs. NSA concentration used in the study is known to effectively inhibit MLKL-dependent necroptosis [3], while HHT concentration used by us was found to effectively inhibit growth of various cell lines [16]. In the case of other assays, cells were typically treated with the indicated drugs at several time points, and the results that were reproducibly obtained at a given time point are shown in the paper. Treatment with SCH 772984, vemurafenib, selumetinib, pictilisib, lapatinib, bafilomycin A1, SBI0206965, SB203580, TAT-Beclin peptide and olaparib was performed as described in the papers cited in the respective sections of the manuscript since under these conditions the drugs were found to effectively induce the intended effects. Concentrations of all drugs used by us are indicated in figure legends.

### Tumor xenografts

Animal studies were approved by Dalhousie University Committee on Laboratory Animals. 6-week-old Nu/Nu Nude mice (Charles River Canada, Saint-Constant, QC) were allowed to acclimatize for 2 weeks. 8 × 10^6^ cells were harvested by trypsin treatment, washed thrice in ice-cold PBS and injected in the muse flank. When tumor volumes reached the average of 80 mm^3^, the mice were injected intraperitoneally (IP) with either DMSO or 4.2 mg/kg NSA or 1 mg/kg HHT or both. NSA was injected daily for 7 days and HHT, daily for 5 days, followed by a 2-day break followed by 5 more days of daily injections. Tumor volumes were measured as published [17].

### Hypodiploid sub-G1 DNA content analysis

20,000 cells/well were seeded in a 6-well plate, harvested after drug treatment using 0.25% trypsin at 37 °C for 5 min, followed by trypsin neutralization with equal volume of the respective serum-containing medium. Cells were pelleted at 500 g for 5 min, washed with 2 ml phosphate-buffered saline (PBS), pelleted at 500 × *g* at 4 °C for 5 min, fixed with 5 ml of 70% ethanol overnight at 4 °C, pelleted at 900 × *g* for 10 min, washed with 3 ml PBS, pelleted at 900 × *g* at 4 °C for 5 min, resuspended in 500 μl of PBS containing 100 μg/mL RNase A, incubated for 30 min at room temperature. 200 µl of propidium iodide was then added to the cells. The cells were analyzed by flow cytometry using BD FACS Celesta instrument, and the data were processed using Flowjo software.

### Assessment of probability of patient’s overall survival

The analysis was carried out by use of GEPIA web tool [18]. The cut off for low and high MLKL mRNA expression levels was 78% and 22%, respectively.

Western blotting [12], detection of clonogenic cell survival [19] and detection of the LC3 puncta [20] were performed as published [19]. Representative images of the cells with the LC3 puncta are shown, and in each case, the scale bar was moved closer to the representative image of the cell from the part of the image not included in the figure. The original western blot images are shown in Supplementary figures.

### Statistical analysis

Statistical analysis of the data in Figs. 1a and 9e was performed by the two-sided chi-square test for goodness-of-fit, and statistical analysis of all other data, by the two-sided Student’s *t* test. The number of times each experiment was repeated with similar results is indicated in the respective figure legends.

## Results

### HHT enhances CRC cell death caused by MLKL inactivation

Oncogenic RAS often drives CRC [21, 22]. We found that RAS-induced activation of a protein kinase ERK promotes CRC cell survival by blocking both apoptotic and non-apoptotic cellular signals [23]. The non-apoptotic events blocked by the RAS/ERK signaling in CRC are understood poorly, and we decided to explore them.

Necroptosis, non-apoptotic programmed cell death driven by plasma membrane permeabilization, is driven by a pseudokinase MLKL which forms an oligomeric structure that disrupts the cell membrane [24, 25]. To test whether ERK inhibition kills CRC by necroptosis we examined whether death of oncogenic RAS-expressing CRC cells HCT116 [26] caused by the ERK inhibitor SCH 772984 [27] is blocked by necrosulfonamide (NSA), a widely used small molecule MLKL inhibitor that binds MLKL at Cys86 [3] and suppresses necroptosis by preventing MLKL incorporation in the plasma membrane [3]. We assessed cell viability by the clonogenic survival assay often utilized for this purpose [23, 28]. We found that NSA did not block SCH 772984-induced cell death (Fig. 1A). Thus, necroptosis inhibition is not the mechanism of ERK-dependent CRC cell survival.

Unexpectedly, even though MLKL typically promotes cell death [24], we found that NSA reduced clonogenic survival of HCT116 cells (Fig. 1A). Of note, *MLKL*-deficient mice are viable, healthy, fertile, are born with normal Mendelian frequency and do not show any physical or behavioral abnormalities [29], i.e. MLKL inactivation is not toxic to the animals. Thus, we decided to test whether MLKL inactivation can potentially serve for CRC treatment. We found that NSA noticeably reduced clonogenic survival of CRC cells LS180 [23], DLD1 [17] and SW480 [30] (Fig. 1B). We further transfected DLD1, one of these cell lines, with a CRISPR/Cas9 vector encoding single guide (sg)RNAs targeting MLKL exons 2 or 9 and generated two cell clones in each of which MLKL gene was disrupted by one of these sgRNAs (Fig. 1C). We found that MLKL-deficient cells are noticeably less clonogenic than the control, MLKL-expressing cells (Fig. 1D). Thus, MLKL inactivation reduces clonogenic survival of CRC cells.

Given that MLKL inactivation reduced CRC cell survival only partially, we searched for approaches enhancing CRC cell death caused by MLKL inactivation. To this end, we tested whether drugs targeting various CRC-promoting mechanisms, e.g. vemurafenib (a RAF inhibitor) [31], selumetinib (a MEK inhibitor) [32], pictilisib (a PI3 kinase inhibitor) [33] or lapatinib [34] (an EGFR/ERBB2 inhibitor) [35], enhance death of DLD1 cells caused by MLKL KO. However, none of the drugs enhanced such death (Fig. 1E). In contrast, a ribosomal inhibitor HHT [5], an alkaloid, also known as omacetaxine, used for treatment of chronic myelogenous leukemia [4, 16], strongly cooperated with MLKL KO in reducing clonogenic survival and the total number of DLD1 cells (Fig. 1E, F). Moreover, HHT and MLKL inhibitor NSA noticeably cooperated in inhibiting clonogenic survival of CRC cells DLD1, HCT116, LS180 and SW480 (Fig. 1G–J). Thus, MLKL inactivation cooperates with HHT in reducing CRC cell survival.

### NSA and HHT cooperate in reducing CRC cell tumorigenicity in vivo

**Fig. 2:**
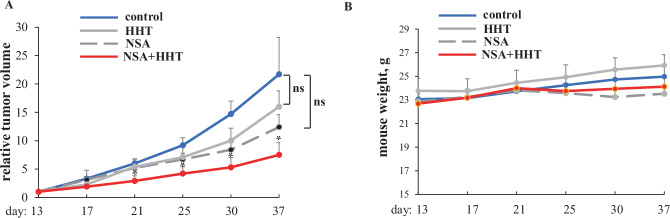
MLKL inhibitor NSA cooperates with HHT in blocking CRC cell tumorigenicity in vivo. **A** HCT116 cells were injected in the flanks of 28 Nu/Nu Nude mice. Once tumor volumes reached the average volume of 80 mm^3^, the mice were injected intraperitoneally with DMSO (control), NSA, HHT or with NSA and HHT together. 7 mice were used per group. One mouse had to be sacrificed in the control (drug-untreated) group on day 25, one more mouse in the same group, on 30 and two more mice in this group, on day 37 because their tumor sizes exceeded 1000 mm^3^ on the respective days. Changes in relative tumor volumes (**A**) and mouse weight (**B**) plus SE are shown. Tumor volume observed on the day of the first injection was designated as 1.0 for each mouse, and subsequent changes in each tumor volume were calculated relative to that number. *p < 0.05.

We further found that NSA and HHT noticeably cooperated in reducing the ability of HCT116 cells, one of these CRC cell lines used by us, to grow as subcutaneous tumors in immunodeficient mice (Fig. 2A). During the treatment, we monitored the mice for weight loss, a widely used surrogate marker of systemic drug toxicity in mouse-based studies [36, 37]. Encouragingly, the mice did not lose weight in response to treatment with each drug alone or both together (Fig. 2B) and did not display any gross physical abnormalities. Thus, NSA and HHT significantly cooperate in suppressing CRC cell tumorigenicity in vivo.

### MLKL does not protect CRC cells from death by secreting soluble factors or by promoting TRAIL receptor degradation

MLKL was suggested to trigger pro-survival signals in certain scenarios, and we tested whether these scenarios apply to our model. It was proposed that MLKL drives secretion of the pro-survival cytokines by breast tumor cells and that these cytokines induce cellular paracrine survival signals [38]. This notion is based on the data showing that cell growth blocked by MLKL KO can be restored by the conditioned medium derived from the parental MLKL-expressing cells [38]. However, we found that MLKL-deficient DLD1 cells cannot be rescued from HHT by the conditioned medium derived from the MLKL-producing cells (Supplementary Fig. 1A). Likewise, the conditioned medium derived from the untreated HCT116 cells, another CRC cell line, did not rescue them from treatment with the combination of NSA and HHT (Supplementary Fig. 1B).

MLKL inactivation was also shown to block lysosomal degradation of the death receptor DR5 in lung and breast tumor cells and thereby enhance cell death upon treatment with TRAIL, the DR5 ligand [39]. Conceivably, DR5 upregulation in MLKL-deficient CRC cells could sensitize the cells to the endogenously produced TRAIL [40, 41]. However, we found that MLKL loss upregulates neither DR5 nor DR4, another TRAIL receptor [2], in CRC cells neither before nor after HHT treatment (Supplementary Fig. 2B, C). Thus, none of the indicated mechanisms mediate the MLKL effect on CRC cells.

### MLKL inactivation and HHT cooperate in killing CRC cells by inhibiting their basal autophagy

Since autophagy is a well-known regulator of cell survival [2], we tested whether MLKL inactivation and HHT treatment cooperate in blocking the basal autophagy in CRC cells. Lipidation of autophagy-driving protein LC3B is a major autophagy mechanism, and formation of LC3B-II, the lipidated LC3B form, is a well-established autophagy marker [42]. LC3B-II is eventually degraded after autophagosome-to-lysosome fusion. Hence, while LC3B-II upregulation can signify increased autophagy, it can also indicate autophagy inhibition caused by reduced fusion of the autophagosome to the lysosome [43]. To ensure that increased LC3B-II levels reflect increased autophagy in our studies, we tested the effect of HHT and MLKL inactivation on LC3B-II in the absence and in the presence of bafilomycin A1, a lysosomal inhibitor that disrupts autophagosome-to-lysosome fusion and thus blocks LC3B-II degradation [43].

**Fig. 3:**
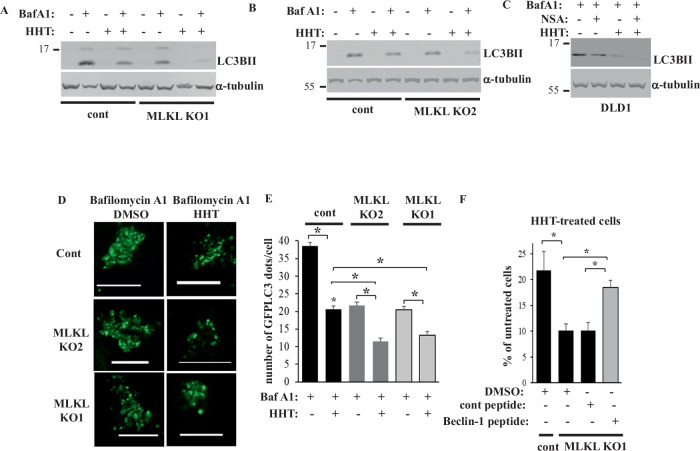
MLKL inactivation and HHT cooperate in killing CRC cells by inhibiting their basal autophagy. **A**, **B** Indicated cell lines were treated with DMSO (−) or 10 ng/ml HHT (+) for 24 h in the absence (−) or in the presence (+) of 50 nM bafilomycin A1, and the cells were assayed for LC3B levels by western blotting. **C** Indicated cell lines were treated with DMSO (−) or 10 ng/ml HHT and/or 3 μM NSA (+) for 24 h in the presence of 50 nM bafilomycin A1 and the cells were assayed for LC3B levels by western blotting. α-tubulin was used as a loading control in **A**–**C**. **D** Indicated cells were transfected with the GFP-LC3 expression vector and treated with DMSO (DMSO) or 10 ng/ml HHT (HHT) for 24 in the presence of 50 nM bafilomycin A1. Green puncta per cell were counted. Representative fluorescence microscopy images are shown. Bar - 10 μm. **E** Quantification of the number of green puncta per cell for the cells treated as in **A**. The numbers represent the average of the number of puncta per cell plus the SE. 24 untreated and 13 HHT-treated cells were counted in the case of the control clone, 42 untreated and 28 HHT-treated cells in the case of MLKL KO1 cells, and 29 untreated and 34 HHT-treated cells, in the case of MLKL KO2 cells This experiment was repeated twice with similar results. **F** Indicated cell lines were treated with DMSO (−) or 10 ng/ml HHT (+) for 96 h in the absence (−) or in the presence (+) of 12 μM of control (cont) or cell-permeable Beclin-1 (Beclin-1) peptide and counted. The data in **F** are the average of three independent experiments plus SD. *p < 0.05.

We found that LC3BII is poorly detectable in bafilomycin A1-untreated cells, and bafilomycin A1 noticeably upregulated LC3BII in DLD1 cells and their MLKL KO variants (Fig. 3A, B). Hence, the basal rate of autophagy in the cells is sufficiently high to trigger LC3BII lysosomal degradation and thereby render LC3BII poorly detectable, unless autophagosome-to-lysosome fusion is blocked. We further observed that MLKL KO, NSA or HHT noticeably downregulated LC3BII in bafilomycin A1-treated MLKL-expressing DLD1 cells (Fig. 3A–C). Remarkably, MLKL KO or NSA significantly cooperated with HHT in downregulating LC3BII (Fig. 3A–C).

To confirm our findings by a complementary approach, we tested the ability of green-fluorescent protein (GFP)-tagged LC3B protein (GFPLC3) to cause puncta formation in the control and MLKL KO CRC cells before and after bafilomycin A1 and HHT treatment. Such puncta are widely used markers of autophagosome formation [43]. Similar to the LC3BII western blot-based data, GFPLC3 puncta were poorly detectable in bafilomycin A1-untreated cells transiently transfected with a GFPLC3-encoding expression vector (not shown) but were readily detectable in bafilomycin A1-treated cells (Fig. 3D, E). We found that MLKL KO and HHT significantly cooperated in reducing the number of the GFPLC3 puncta per cell (Fig. 3D, E). Thus, MLKL inactivation and HHT cooperate in reducing the basal CRC cell autophagy.

To test whether autophagy inhibition triggered by HHT in MLKL-deficient cells is the cause of CRC cell loss, we treated the MLKL KO cells with HHT and the cell permeable peptide composed of the HIV-1 TAT protein transduction domain attached to either a scrambled amino acid sequence (control peptide) or the peptide derived from a protein Beclin-1, a major autophagy inducer that promotes autophagy by activating class III PI3 kinase (PI3K) [44]. The TAT domain renders the peptide cell-permeable while the respective Beclin 1 fragment is necessary and sufficient for autophagy induction [44]. The peptide is being widely used as an autophagy-promoting tool [44–47]. Similar to Beclin 1, the peptide increases Class III PI3K complexes kinase activity and induces autophagy in a manner that requires the presence of autophagy drivers ATG 5 and ATG7 [47]. We found that the Beclin-1-derived peptide almost completely prevented MLKL KO from reducing the growth of HHT-treated DLD1 cells (Fig. 3F). Thus, autophagy inhibition caused by MLKL inactivation combined with HHT is required for CRC cell death induced by this combination treatment. In light of this, our findings that RAF, MEK, PI3K and EGFR inhibitors did not cooperate with MLKL in killing CRC cells (Fig. 1E) are not surprising since these agents promote, rather than inhibit, autophagy in various cancer cell types, including CRC cells [48–50].

**Fig. 4:**
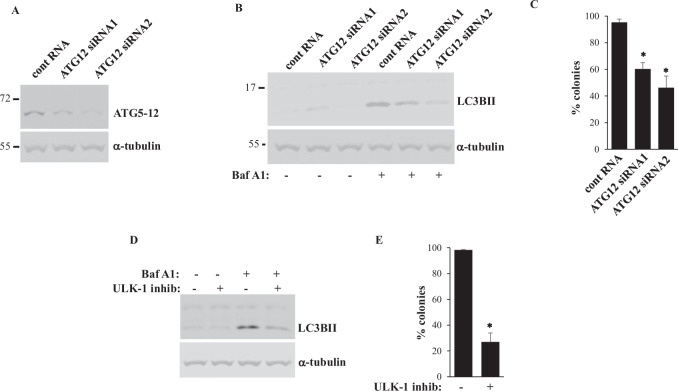
Basal autophagy inhibition is suffcieent for reducing CRC cell clonogenic survival. **A** DLD1 cells were transfected with a 100 nM control RNA (cont RNA) or ATG12-specidic siRNA (ATG12 siRNA) 1 or 2 and assayed for the levels of the covalent ATG5-ATG12 complex (ATG5-12) by western blotting. **B** Cells treated as in **A** and cultured in the absence (−) or in the presence (+) of 50 nM bafilomycin A1 were assayed for LC3BII levels by western blotting. **C** Cells treated as in **A** were assayed for clonogenic survival. **D** DLD1 cells were treated with DMSO (−) or 20 μM ULK-1 inhibitor (ULK-1 inhib) SBI-0206965 (+) for 24 h in the absence (-) or in the presence (+) of 50 nM bafilomycin A1 were assayed for LC3BII levels by western blotting. **E** DLD1 cells were treated as in **D** for 96 h and counted. α-tubulin was used as a loading control in **A**, **B**, **D**. The data in **C**, **E** are the average of three independent experiments plus SD. *- *p* < 0.05.

We further knocked down (KD) ATG12, a major autophagy driver [42], in DLD1 cells by two different siRNAs. Of note, ATG12 forms a covalent complex with a protein ATG5 to promote LC3 lipidation, and ATG12 knockdown downregulated this complex (Fig. 4A). Furthermore, ATG5-ATG12 KD or treatment with SBI0206965, a specific inhibitor of the critical autophagy driver protein kinase ULK1 [51], noticeably reduced LC3 lipidation in Bafilomycin A1-treated cells (Fig. 4B, D) and suppressed growth of MLKL-expressing DLD1 cells (Fig. 4C, E). Remarkably, the more efficient ATG5-ATG12 KD was, the more efficiently LC3B lipidation and cell growth were inhibited (Fig. 4A–C). Thus, inhibition of autophagy is sufficient for suppressing CRC cell growth. These data are consistent with observations that autophagy inhibition substantially reduces growth of colorectal cancer patient-derived xenografts and multiple human colorectal cancer cell lines [52–58]. In summary, we conclude that MLKL inactivation and HHT cooperate in killing CRC cells by inhibiting their basal autophagy.

### MLKL inactivation renders CRC cell autophagy VPS37A-dependent

**Fig. 5:**
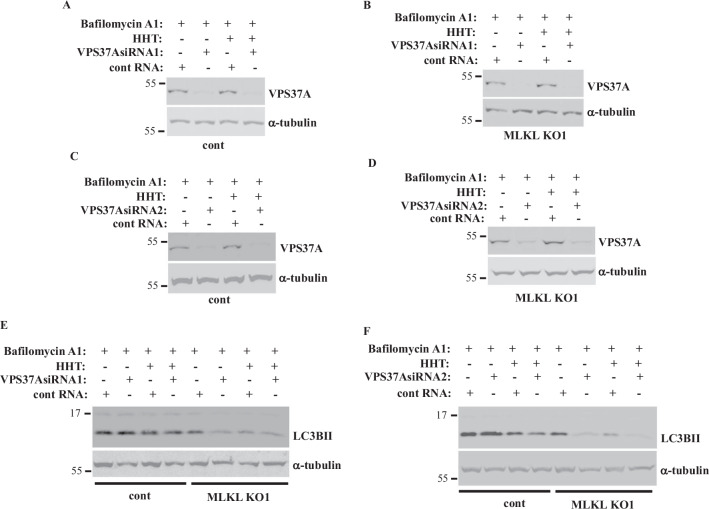
**MLKL inactivation renders CRC cell autophagy VPS37A-dependent**. Indicated cell lines were transfected with a 100 nM control RNA (cont RNA) or VPS37A-specidic siRNA (VPS37A siRNA) 1 (**A**, **B**, **E**) or 2 (**C**, **D**, **F**), treated with DMSO (−) or 10 ng/ml HHT (+) for 24 h in the presence of 50 nM bafilomycin A1 and assayed for VPS37A (**A**–**D**) or LC3B (**E**, **F**) levels by western blotting. α-tubulin was used as a loading control.

The ESCRT machinery includes the ESCRT-I, -II and III multiprotein complexes that promote the formation of various vesicles, such as endosomes and autophagosomes [8]. During necroptosis, MLKL was found to activate ESCRT-I and III which repaired the MLKL-permeabilized cell membrane and delayed cell death [59]. To examine the role of ESCRT-I in the effects observed by us, we studied LC3BII levels in bafilomycin A1-treated MLKL-expressing and MLKL KO DLD1 cells before and after KD of the critical ESCRT-I complex component VPS37A by two different VPS37A-specific siRNAs (Fig. 5A–D). Notably, VPS37A KD did not have any effect on LC3B lipidation of the control, MLKL-expressing DLD1 cells but strongly reduced LC3B lipidation in MLKL KO DLD1 cells (Fig. 5E, F). Hence, such lipidation becomes VPS37A-dependent in the absence of MLKL. Moreover, VPS37A was unable to promote/support LC3B lipidation in MLKL-deficient HHT-treated cells (Fig. 5E, F). Thus, MLKL inactivation reduces CRC cell autophagy and renders this autophagy VPS37A-dependent. However, VPS37A is unable to promote autophagy of HHT-treated cells.

### HHT contributes to autophagy inhibition in CRC cells by activating p38MAPK

**Fig. 6:**
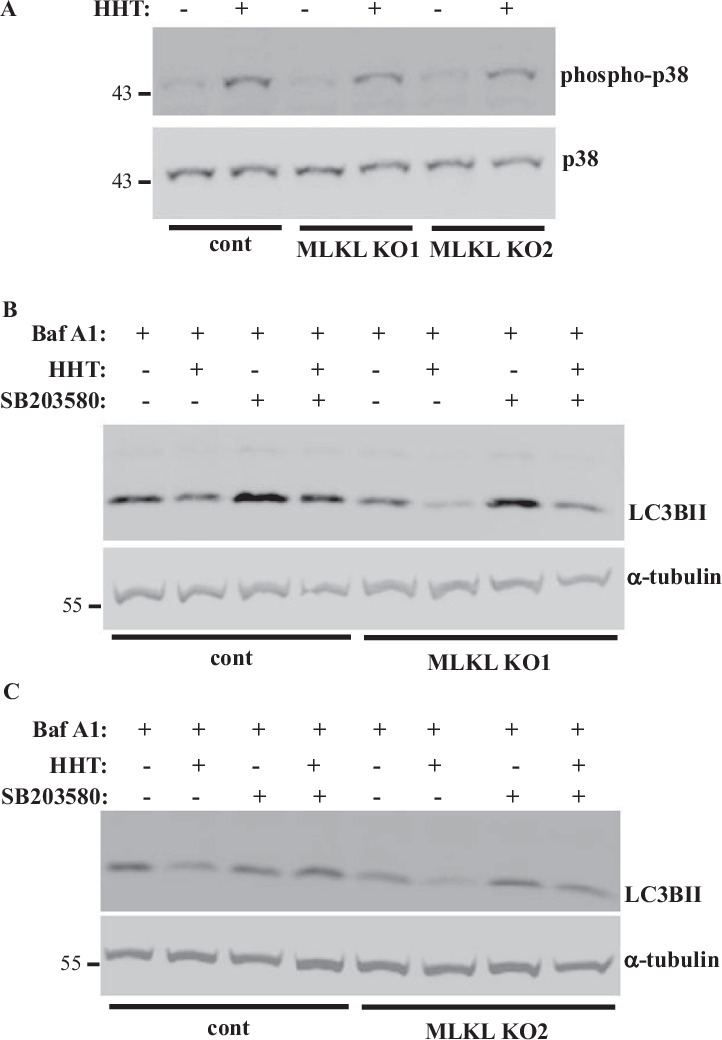
**HHT contributes to autophagy inhibition in CRC cells by activating p38MAPK**. Indicated cell lines were treated with DMSO (−) or 10 ng/ml HHT for 24 h and/or 20 μM p38MAPK inhibitor SB203580 (SB203580) (+) in the presence of 50 nM bafilomycin A1 and assayed for p38MAPK and phospho-38MAPK (**A**) or LC3B (**B**, **C**) levels by western blotting. α-tubulin was used as a loading control in **B** and **C**.

HHT is a ribosome inhibitor, and various ribosome inhibitors cause ribotoxic stress response, a chain of signals triggered by the ribosome-bound protein kinase ZAKα. ZAKα further phosphorylates and activates MAP kinases MKK3 and/or MKK6 which in turn phosphorylate and activate p38MAPK and JNK [6]. Notably, p38MAPK inhibits autophagy via multiple mechanisms, including phosphorylation and inactivation of the autophagy stimulators ULK1 [60] and ATG5 [61] as well as inactivation of the autophagy stimulator ATG9 [62]. We found that HHT upregulates phospho-p38MAPK, the sign of p38MAPK activation [63, 64], in both the control and MLKL KO DLD1 cells (Fig. 6A). Four members of the p38MAPK family are known, α, β, γ and δ[64]. We found that treatment of the MLKL KO DLD1 cells with SB203580, a specific small molecule p38MAPK α and β inhibitor [65], in the presence of Bafilomycin A1 rendered their LC3BII levels comparable to those observed in the untreated cells (Fig. 6B, C). Hence, HHT-induced p38MAPK activation likely mediates HHT-induced autophagy inhibition in MLKL-deficient CRC cells.

### MLKL inactivation and HHT cooperate in killing CRC cells by parthanatos

**Fig. 7:**
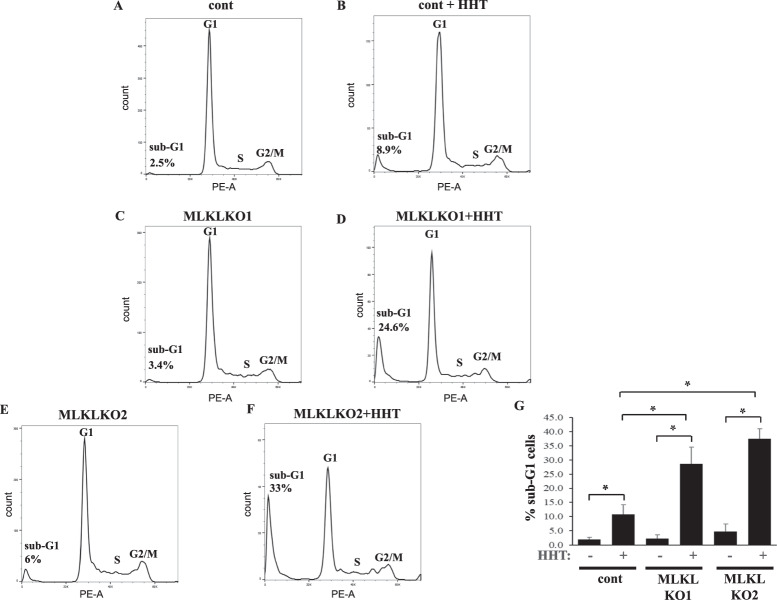
MLKL inactivation and HHT cooperate in triggering chromosomal DNA fragmentation in CRC cells. Indicated cell lines were treated with with 10 ng/ml HHT (HHT) or DMSO for 96 h and analyzed for the cell cycle profile by flow cytometry. **A**–**F** Representative cell cycle profiles are shown. **G** The average % of the hypodiploid sub-G1 cells for the indicated treatments derived from three independent experiments plus SD is shown. *p < 0.05.

We examined which of the known cell death forms [2] is triggered by the combined inactivation of MLKL and HHT treatment. When using flow cytometry to analyze the distribution of CRC cells between the phases of the cell cycle based on their DNA content, we noticed that MLKL KO and HHT cooperated in increasing the population of cells with the hypodiploid sub-G1 DNA content (Fig. 7A–G). The presence of such population indicates chromosomal DNA fragmentation [66], a sign of regulated cell death types, e.g., apoptosis and parthanatos [2]. When we cultured the MLKL KO DLD1 cells with HHT and the inhibitor of caspases, apoptosis-executing proteases, zVAD-FMK [67], this caused massive chromosomal DNA degradation regardless of MLKL presence (not shown). Notably, caspase inhibition was found to trigger non-apoptotic cell death in multiple studies [68, 69]. Since the ability of HHT to kill cells strongly depends on the presence of MLKL (Fig. 1), we concluded that HHT treatment and MLKL inactivation unlikely cooperate in causing CRC cell apoptosis.

Parthanatos is programmed necrosis triggered by hyperactivation of poly(ADP-ribose) polymerase 1 (PARP1), which drives the formation of poly(ADP-ribose) (PAR) [11]. When overproduced, PAR causes the release of the protein AIF from the mitochondria [11]. AIF then binds the endonuclease MIF, and the AIF–MIF complex triggers chromosomal DNA degradation and cell death [11].

**Fig. 8:**
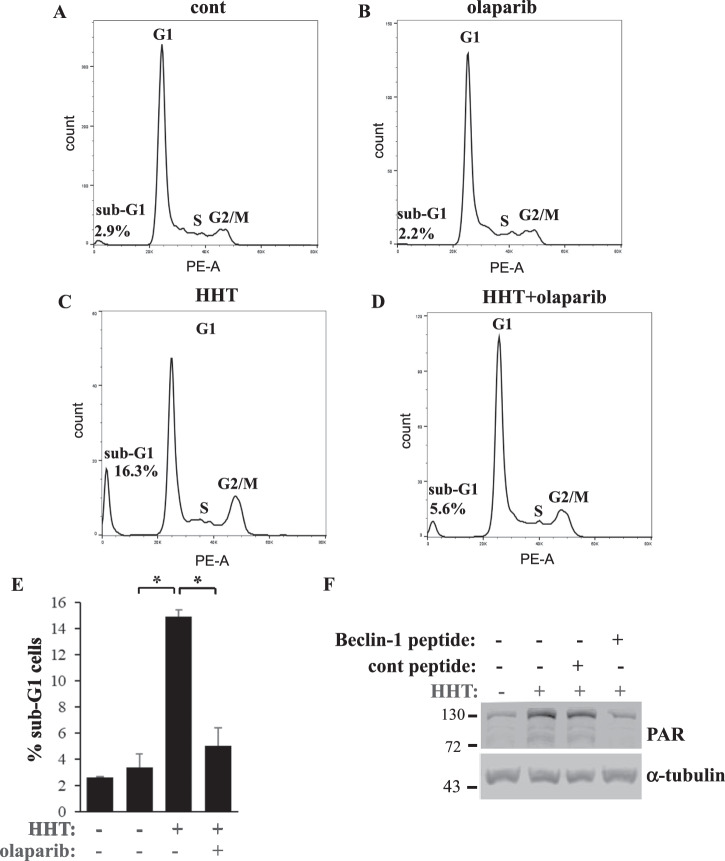
MLKL inactivation and HHT cooperate in triggering parthanatos of CRC cells. MLKL KO1 cells lines were treated with DMSO (cont), 10 ng/ml HHT (HHT), 50 nM olaparib (Olaparib), or both drugs (HHT+ Olaparib) at the indicated concentrations for 96 h and analyzed for the cell cycle profile by flow cytometry. **A**–**D** Representative cell cycle profiles are shown. **E** The average % of the hypodiploid sub-G1 cells for the indicated treatments derived from three independent experiments plus SD is shown. *p < 0.05. **F** MLKL KO1 cells lines were treated with DMSO (−) or 10 ng/ml HHT (+) in the absence (−) or in in the presence (+) of 12 μM of control (cont) or cell-permeable Beclin-1 (Beclin-1) peptide for 96 h and analyzed for poly(ADP-ribose) (PAR) levels by western blotting. α-tubulin was used as a loading control.

We found that HHT-induced formation of the sub-G1 DNA content in the MLKL KO DLD1 cells is prevented by the PARP inhibitor olaparib [70] (Fig. 8A–E). Moreover, HHT caused a significant increase in the multiple species of PAR polymers in the cells, a parthanatos symptom (Fig. 8F) [71]. Furthermore, the autophagy-inducing Beclin1-derived cell-permeable peptide [44] prevented HHT from upregulating PAR in the cells (Fig. 8F). Hence, MLKL inactivation combined with HHT treatment cooperates in blocking CRC cell autophagy and this autophagy inhibition triggers parthanatos.

### Low MLKL mRNA expression signifies increased CRC patient survival

**Fig. 9:**
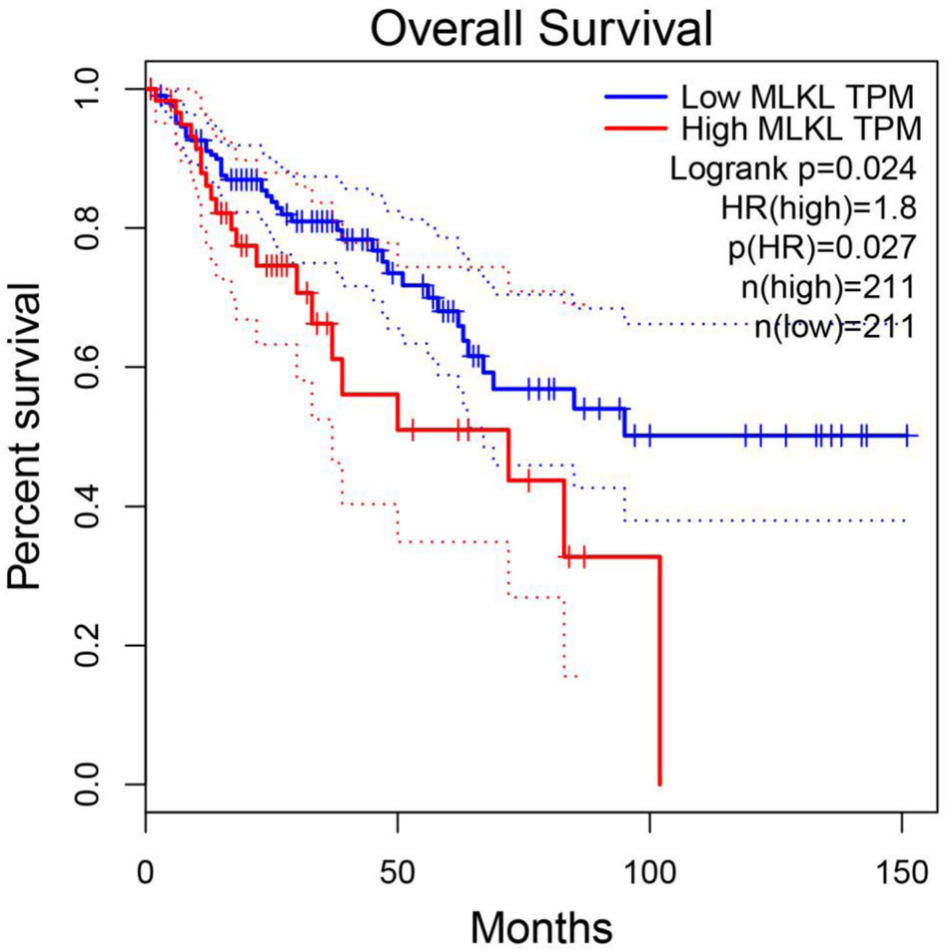
Low MLKL mRNA expression signifies increased CRC patient survival. Kaplan–Meier analysis-based estimation of probabilities of patients’ survival depending on the level of MLKL mRNA was performed.

To test whether MLKL expression correlates with CRC patient survival we used GEPIA, an interactive web server for analyzing the RNA sequencing expression data in various tumors derived from the TCGA and the GTEx projects [18]. We found that reduced levels of MLKL mRNA in CRC correlate with increased patient survival (Fig. 9). These data are consistent with observations indicating that reduced levels of phosphorylated, i.e., activated, MLKL protein in CRC and esophageal tumors correlate with increased survival of the respective patients [38]. Collectively, these results support the notion that the lower the levels of active MLKL expression in CRC cells are, the less viable the cells are, the less deadly respective cancers become, the longer patients survive.

In summary, we have shown here that pharmacological or genetic MLKL inactivation, when combined with HHT, blocks the basal CRC cell autophagy and triggers parthanatos of CRC cells. Moreover, a pharmacological MLKL inhibitor and HHT cooperate in suppressing CRC cell tumorigenicity in vivo. Hence, MLKL inhibition combined with HHT treatment is a potential novel approach for CRC therapy.

## Discussion

We found that MLKL inactivation when combined with a clinically approved anti-cancer drug HHT represents an effective approach for killing CRC cells in vitro and suppressing their tumorigenicity in vivo. Hence, combining MLKL inactivation with HHT treatment is a potential novel approach to CRC management.

MLKL drives cell death during necroptosis [2]. We found that MLKL also promotes CRC cell survival by supporting the basal CRC cell autophagy. We discovered that MLKL inactivation and HHT treatment cooperate in blocking such autophagy and killing CRC cells. Our data indicate that MLKL mediates LC3B lipidation, a critical autophagy step, in CRC cells. The ESCRT-I complex is known to promote autophagy [10] but apparently, the ESCRT-I component VPS37A is not needed for driving LC3 lipidation in MLKL-expressing CRC cells. However, in MLKL-deficient cells, LC3B lipidation is strongly reduced and becomes almost entirely VPS37A-dependent. Notably, so far VPS37A was found to drive autophagy by supporting the autophagosomal membrane integrity and/or promoting the closure of this membrane [9, 10]. To our knowledge, the role of the ESCRT-I machinery in LC3 lipidation has never been demonstrated, which is not surprising since this role becomes apparent only in the absence of MLKL, a scenario that has not been tested so far. Understanding how VPS37A controls such lipidation and how this process is regulated by MLKL is the subject of our ongoing studies.

We have shown here that the ability of MLKL antagonists to suppress CRC cell autophagy is enhanced by HHT and that this effect is driven by HHT-induced p38MAPK activation. HHT is a ribosome inhibitor [5], and such inhibitors often activate p38MAPK [6]. Our data are consistent with these findings and with observations that p38MAPK inhibits autophagy by multiple mechanisms [60–62].

Since both NSA and HHT inhibit autophagy, would it be possible to use either drug alone at a higher dose to suppress autophagy and thereby suppress CRC in the clinic? We found that even complete MLKL loss has only a limited effect on CRC cell autophagy and survival. Moreover, while HHT is effective against CML [4], the use of HHT as a single agent at non-toxic doses was ineffective against CRC in the clinic [72], and a higher HHT dose was unacceptably toxic [73]. Thus, the use of MLKL antagonists could render the non-toxic HHT doses sufficient for reducing the basal autophagy in CRC cells below the levels required for cell survival and thereby make this combination treatment efficacious against CRC.

We found that autophagy inhibition caused by MLKL inactivation and HHT treatment kills CRC cells by parthanatos. Oxidative stress and excessive DNA damage are the known consequences of autophagy inhibition in cancer cells [74, 75] and represent the established stimuli of parthanatos [11]. Testing whether these stimuli trigger parthanatos in CRC cells in response to MLKL inactivation and HHT treatment represents a promising direction for our future studies.

We found that an MLKL inhibitor NSA [3] strongly cooperates with HHT in suppressing growth of tumors formed by CRC cells in mice. Thus, pharmacological MLKL inhibition combined with HHT treatment is a potential novel approach for CRC management. Importantly, PARP1, a major parthanatos inducer, is overexpressed in human tumor-derived colon cancer cells compared to normal intestinal epithelium and promotes colon cancer in mice [76]. Conceivably, high PARP1 level in CRC cells could make them vulnerable to therapies based on MLKL inhibition combined with the use of HHT, and thus create a therapeutic window for CRC treatment. Therefore, PARP1 could be explored as a biomarker of CRC sensitivity to this combination treatment.

## Supplementary information

Supplementary figures

Original western blots

## Data Availability

We will share all data reported in this study upon request.
