# Supplementary material for: Inactivation of necroptosis-promoting protein MLKL creates a therapeutic vulnerability in colorectal cancer cells
Source: Cell Death Dis. 2025 Feb 20;16(1):118. doi: 10.1038/s41419-025-07436-z (PMC11842741; doi:10.1038/s41419-025-07436-z)
Supplement: Supplementary file 1 — Supplementary figures [file 41419_2025_7436_MOESM1_ESM.docx]

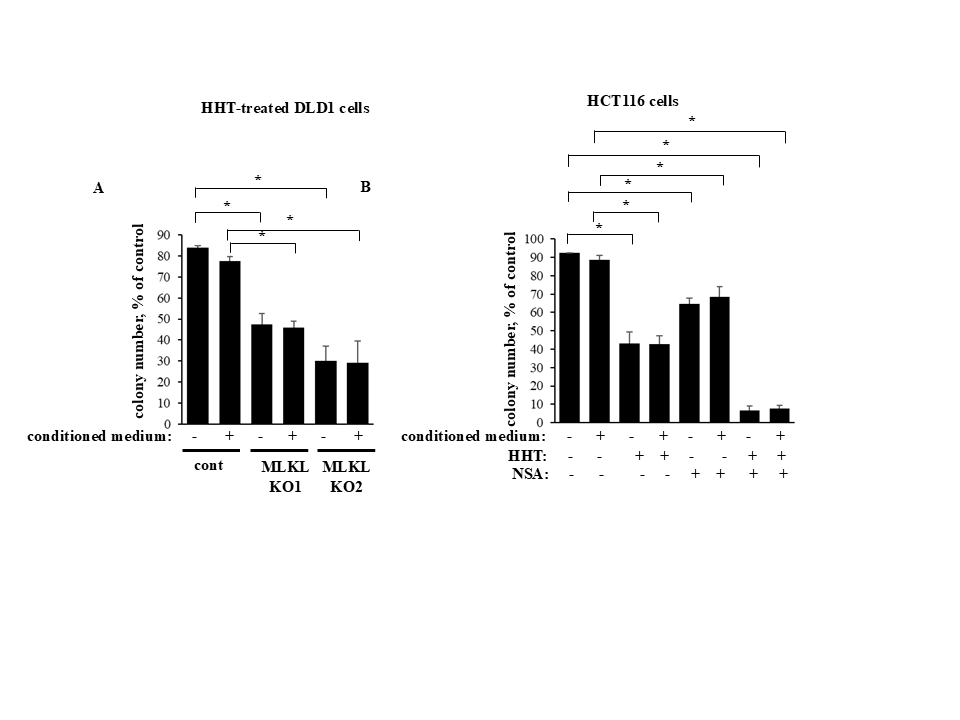


Supplementary Fig. 1 MLKL does not protect CRC cells from death by secreting soluble factors. (**A**) Indicated cell lines were treated (+) or not (-) with 10 ng/ml HHT in the absence (-) or in the presence (+) of the conditioned medium derived from the same number of control untreated cells, and colonies formed by the cells were counted. (**B**) HCT116 cells were treated (+) or not (-) with 10 ng/ml HHT and/ot 1μM NSA in the absence (-) or in the presence (+) of the conditioned medium derived from the same number of control untreated cells, and colonies formed by the cells were counted. The data in (**A, B**) are the average of three independent experiments plus SD. * *p* value < 0.05.


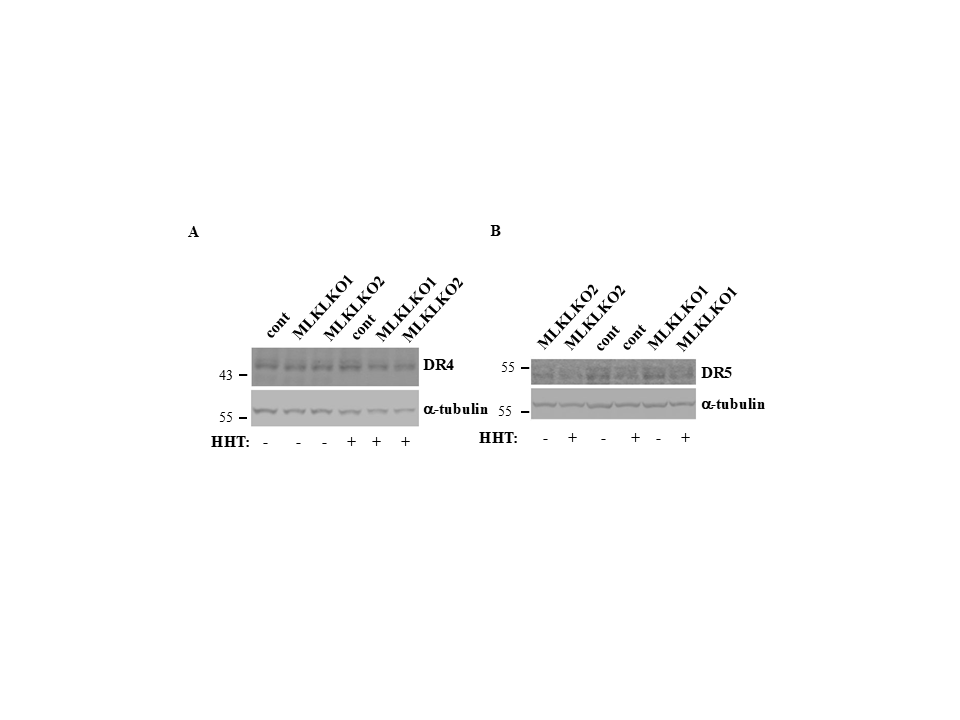


Supplementary Fig. 2 MLKL does not promote TRAIL receptor degradation. Indicated cell lines were treated with 10 ng/ml HHT for 72h (**A**) or 48h (**B**) and assayed for DR4 (**A**) or DR5 (**B**) levels by western blotting. α-tubulin was used as a loading control.
