# Supplementary material for: Inactivation of necroptosis-promoting protein MLKL creates a therapeutic vulnerability in colorectal cancer cells
Source: Cell Death Dis. 2025 Feb 20;16(1):118. doi: 10.1038/s41419-025-07436-z (PMC11842741; doi:10.1038/s41419-025-07436-z)
Supplement: Supplementary file 2 — Original western blots [file 41419_2025_7436_MOESM2_ESM.docx]

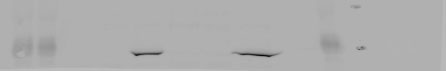


**MLKL**


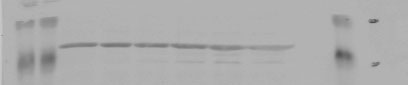


**GAPDH**

Supplementary fig. 3 Original western blots for the data shown in Fig. 1C. Lanes 3-1 are shown in Fig. 1C.





**DR4**


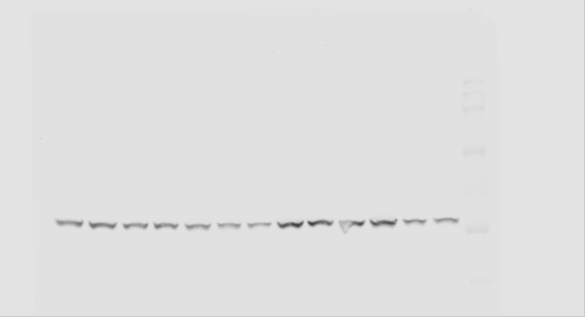


**α-tubulin**

Supplementary fig. 4 Original western blots for the data shown in Supplementary Fig. 2A. Lanes 2-7 are shown in Supplementary Fig. 2A.





**DR5**





**α-tubulin**

Supplementary fig. 5 Original western blots for the data shown in Supplementary Fig. 2B. Lanes 5-10 are shown in Supplementary Fig. 2B.





**LC3BII**





**α-tubulin**

Supplementary fig. 6 Original western blots for the data shown in Fig. 3A. Lanes 7-14 are shown in Fig. 3A





**LC3BII**


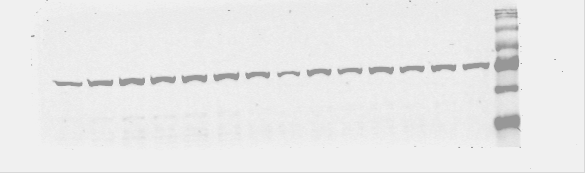


**α-tubulin**

Supplementary fig. 7 Original western blots for the data shown in Fig. 3B. Lanes 9-16 are shown in Fig. 3B.


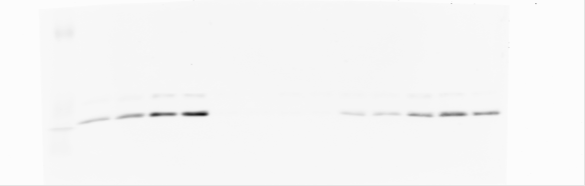


**LC3BII**


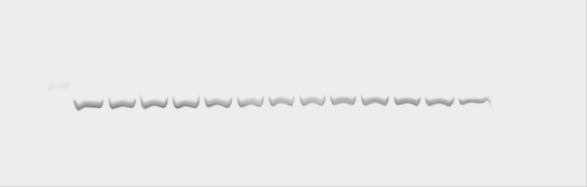


**α-tubulin**

Supplementary fig. 8 Original western blots for the data shown in Fig. 3C. Lanes 10-14 are shown in Fig. 3C


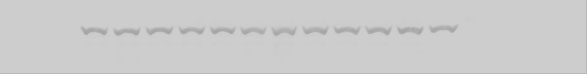

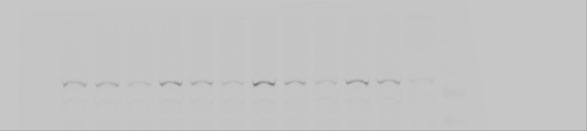
Supplementary fig. 9 Original western blots for the data shown in Fig. 4A. Lanes 7-10 are shown in Fig. 4A.

**α-tubulin**

**ATG12**


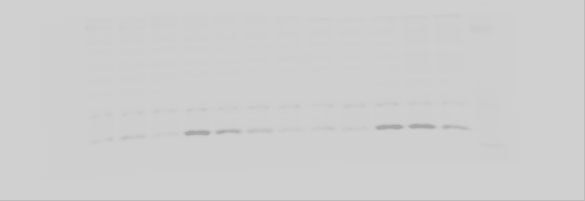


**LC3BII**


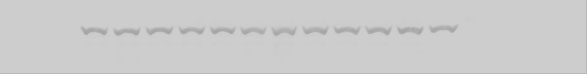


**α-tubulin**

Supplementary fig. 10 Original western blots for the data shown in Fig. 4B. Lanes 1-6 are shown in Fig. 4B.


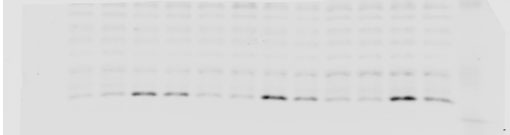


**LC3BII**


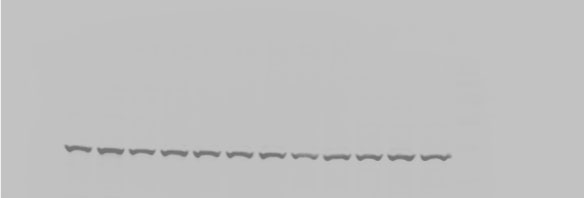


**α-tubulin**


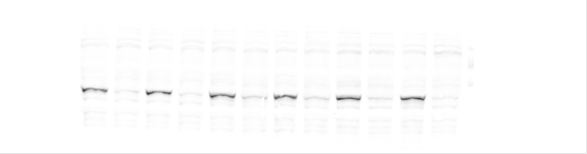
Supplementary fig. 11 Original western blots for the data shown in Fig. 4D. Lanes 9-12are shown in Fig. 4D.

**VPS37A**





**α-tubulin**

Supplementary fig. 12 Original western blots for the data shown in Fig. 5A, B. Lanes 5-8 are shown in Fig. 5A and lanes 1-4 are shown in Fig. 5B.





**VPS37A**





**α-tubulin**

Supplementary fig. 13 Original western blots for the data shown in Fig. 5C. Lanes 5-8 are shown in Fig. 5C





**VPS37A**





**α-tubulin**

Supplementary fig. 14 Original western blots for the data shown in Fig. 5D. Lanes 1-4 are shown in Fig. 5D.


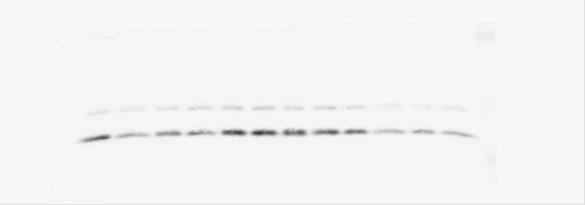


**LC3BII**


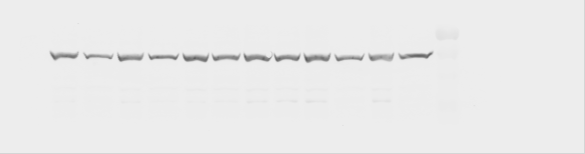


**α-tubulin**

Supplementary fig. 15 Original western blots for the data shown in Fig. 5E. Lanes 5-12 are shown in Fig. 5E.


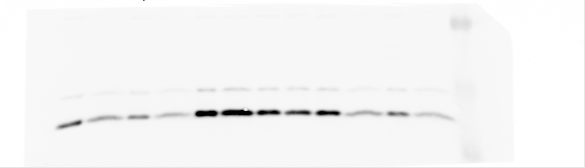


**LC3BII**


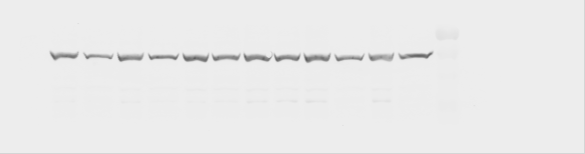


**α-tubulin**

Supplementary fig. 16 Original western blots for the data shown in Fig. 5F. Lanes 5-12 are shown in Fig. 5F.





**pp38**





**p38**

Supplementary fig. 17 Original western blots for the data shown in Fig. 6A. Lanes 7-12 are shown in Fig. 6A.





**LC3BII**


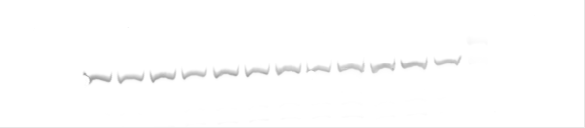


**α-tubulin**

Supplementary fig. 18 Original western blots for the data shown in Fig. 6B. Lanes 5-12 are shown in Fig. 6B.


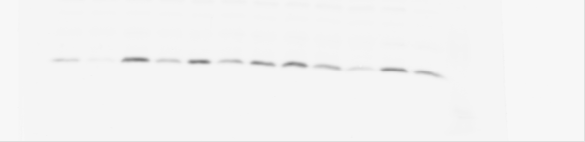


**LC3BII**





**α-tubulin**

Supplementary fig. 19 Original western blots for the data shown in Fig. 6C. Lanes 5-12 are shown in Fig. 6C.





**PAR**


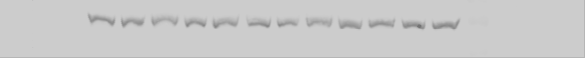


**α-tubulin**

Supplementary fig. 20 Original western blots for the data shown in Fig. 8F. Lanes 5-8 are shown in Fig. 8F.
